# How to choose appropriate direct oral anticoagulant for patient with nonvalvular atrial fibrillation

**DOI:** 10.1007/s00277-015-2566-x

**Published:** 2015-12-11

**Authors:** Jordan K. Schaefer, Robert D. McBane, Waldemar E. Wysokinski

**Affiliations:** Department of Internal Medicine, Mayo Clinic, Rochester, MN USA; Division of Cardiovascular Diseases, Mayo Clinic and Foundation for Education and Research, 200 1st Street SW, Rochester, MN 55905 USA; Division of Hematology Research, Mayo Clinic, Rochester, MN USA

**Keywords:** Direct thrombin inhibitors, Factor Xa inhibitors, Rivaroxaban, Dabigatran, Apixaban, Warfarin, Nonvalvular atrial fibrillation

## Abstract

The novel oral anticoagulants or direct oral anticoagulants (DOAC) are becoming more common in clinical practice for the prevention of stroke in non-valvular atrial fibrillation (NVAF). The availability of several agents with similar efficacy and safety for stroke prevention in NVAF patients offers more selection, but at the same time requires certain knowledge to make a good choice. This comparative analysis provides an appraisal of the respective clinical trials and highlights much of what remains unknown about four FDA-approved agents: dabigatran, apixaban, rivaroxaban, and edoxaban. It details how the DOACs compare to warfarin and to one another summarizes pharmacologic and pharmacodynamic properties, and drug interactions from the stand point of practical consequences of these findings. Common misconceptions and reservations are addressed. The practical application of this data is intended to help choosing the most appropriate agent for individual NVAF patient.

## Introduction

In 2010, the FDA approved dabigatran for stroke prevention in patients with non-valvular atrial fibrillation (NVAF) and ended a long era of vitamin K antagonists (VKA) as the mainstay of oral anticoagulation for this indication [1]. Within the last 5 years, four direct oral anticoagulants (DOACs): dabigatran, rivaroxaban, apixaban, and edoxaban, were assessed in large phase III clinical trials, compared to either warfarin [2–5] or aspirin [6], for stroke prevention in patients with NVAF. Results of these trials have led to FDA approval of these agents, along with endorsement by a growing number of guidelines and regulatory bodies worldwide [7–20]. Although there are abundant data on DOAC efficacy and safety compared to warfarin, and a growing experience on their interaction with other medications, there is a general lack of knowledge of the application of these findings into clinical practice. In the end, choosing the most appropriate DOAC for an individual NVAF patient remains complicated. This review is intended to concentrate on useful practical implications of our knowledge about DOACs (formerly known as novel anticoagulants NOACs), and to resolve some misconceptions, and reservations regarding this group of anticoagulants to help in every day application of these agents.

## Background

VKAs inhibit the carboxylation of all vitamin K-dependent procoagulant factors II, VII, IX, X, but also have the “off target” effect of inhibiting the natural anticoagulants, protein C, and protein S. In contrast, DOACs target specific proteins in the coagulation cascade. These targeted factors were well chosen given their central participation in the coagulation cascade. Factor Xa serves as the convergent enzyme where the intrinsic and extrinsic pathways meet to form the final common pathway of prothrombin activation. It is estimated that one molecule of factor Xa is responsible for generating more than 1000 thrombin molecules [21]. Direct factor Xa inhibition (rivaroxaban, apixaban, edoxaban) turns off prothrombin activation upstream, thus limiting available thrombin. Moreover, unlike low molecular weight heparin or pentasaccharide therapy, direct factor Xa inhibitors have access to factor Xa sequestered within the prothrombinase complex. Targeting thrombin by dabigatran is also quite logical, as thrombin cleaves fibrinogen to fibrin as the final step of clot formation. Thrombin activates factor XIII, which stabilizes the developing clot. Moreover, thrombin self-amplifies its own generation by activating factors V and VIII, and is the most potent platelet activator.

## General characteristics

There are several characteristics which distinguish DOACs, as a group, from VKAs. First, they have rapid onset of action (1–3 h) and, consequently, do not require “bridging” with parenteral anticoagulants. Second, there is no need for routine monitoring of anticoagulation. Third, they have similar (7–15 h) half-lives. Fourth, they are all, to some extent, partially eliminated by the kidney: 80 % of dabigatran, 50 % of edoxaban, 35 % of rivaroxaban, and 25 % of apixaban [5, 17]. Patients with impaired kidney function should therefore use these medications with caution. Despite similar elimination kinetics, the dosing frequency differs between the agents; dabigatran and apixaban are dosed twice daily while rivaroxaban and edoxaban are given once a day. Patients with busy schedule or compliance concerns might do better with agents dosed once a day. A summary of characteristics with meaningful, practical implications are provided (Table 1).

**Table 1:** General characteristic of direct oral anticoagulants.

Characteristics	Dabigatran	Rivaroxaban	Apixaban	Edoxaban
Renal clearance (%)	80	35	25	50
Bioavailability (%)	pH dependent^a^ 6–7	Food dependent^b^ 66–≥80	Food independent 50	Food independent 62
Medication storage	In original bottle or blister package	Room temperature	Room temperature	Room temperature
Liver metabolism: CYP3A4 metabolism	No	Yes	Minor	Minor
Impacted by P-glycoprotein transporter system	Yes	Yes	Yes	Yes

^a^Ten milligram dose has oral bioavailability independent of food but 15 and 20 mg doses of rivaroxaban have achieved high bioavailability (≥80 %) when taken with food

^b^Tartaric acid added into the dabigatran capsule to ensure optimal and gastrointestinal pH independent absorption is responsible for 5–10 % incidence of dyspepsia

Providers should be aware of the characteristics that distinguish DOACs from one another. For example, only 6–7 % gastrointestinal absorption of dabigatran implies that slight fluctuations in absorption or elimination may have a profound impact on plasma levels. To reduce the impact of varying gastrointestinal acidity on pro-drug absorption, dabigatran is formulated with tartaric acid [22]. Therefore, breaking, chewing, or emptying the contents of the capsule is prohibited and the patients are instructed to swallow the capsule whole. Moreover, dabigatran capsules are susceptible to ambient moisture. Consequently, medication storage must be in the original bottle or blister package until use. The tartaric acid spherules may be responsible for a 5–10 % incidence of dyspepsia [2]. Patients with peptic ulcer disease, subtotal or total gastrectomy, or gastric bypass surgery should rather avoid dabigatran or use it with caution. It is also noteworthy that rivaroxaban at 15 and 20 mg doses must be taken with food because of higher bioavailability (from 66 to more than 80 %) [23]. The other DOACs do not have this requirement even though edoxaban has 6–22 % better [24] absorption when taken with food. Apixaban and rivaroxaban have similar bioavailability when administered in crushed form and therefore can be administered via a nasogastric tube [25]; no data are available yet on bioavailability of crushed tablets of edoxaban.

Although less frequent than warfarin, there are important drug interactions to consider. There are two main mechanisms by which drug interactions occur with DOACs which are clinically relevant (Table 1). The P-glycoprotein (P-gp) transporter system serves to secrete medication back into the intestinal lumen, bile, and urine, thus reducing drug exposure [26]. Medications which induce this system will, thereby, reduce drug exposure. Conversely, competitive inhibition will increase drug exposure. The hepatic cytochrome p450 CYP3A4 is the other important pathway in the metabolism of all DOACs, with the exception of dabigatran [20, 23, 24]. Medications that impact both P-gp and CYP3A4 pathways may influence the effect of DOACs [17–24]. Strong inducers to remember include carbamazepine, phenytoin, rifampin, St John’s wort, and tipranavir/ritonavir which decrease circulating DOAC levels. Strong inhibitors include itraconazole, ketoconazole, lopinavir/ritonavir, indinavir, and voriconazole which increase DOAC levels. Such drugs should generally not be co-administered with DOACs [20, 26]. For apixaban, most of the hepatic clearance occurs without metabolism [27]. Therefore, the US package insert recommends reducing the dose of apixaban from 5 to 2.5 mg twice daily when co-administered with a strong CYP3A4 and P-gp inhibitors and to avoid apixaban use if the patient already requires a dose reduction due to other clinical cofounders (age >80, weight <60 kg, creatinine >1.5). The European package insert recommends avoiding apixaban use with a strong CYP3A4 and P-gp inhibitors. While changes in CYP3A4 do not appear to greatly impact edoxaban metabolism, caution is warranted until more definitive interaction data are available [28].

Information on dose adjustment and interaction of DOACs with commonly used cardiovascular medications in patients with NVAF are important to review (Table 2). Therapy should be tailored for each patient, not only taking into account coexisting medications but also comorbidities, including renal and liver function, general bleeding risk, and propensity for thrombosis.

**Table 2:** Interaction of direct oral anticoagulants with commonly used cardiovascular medications

Medications	Dabigatran	Rivaroxaban	Apixaban	Edoxaban
Quinidine	Use with caution^a^	Use with caution^a^	No data	Use with caution^a^
Verapamil	Use with caution^a, b^	Minor effect	No data	Use with caution^a^
Amiodarone	Use with caution^a^	Use with caution^a^	No data	Use with caution^a^
Dronedarone	Avoid	Use with caution^a^	No data	50 % dose reduction^c^
Ranolazine	No effect	Use with caution^a^	No data	No data
Digoxin	No effect	Minimal effect	No effect	Minimal effect
Atorvastatin	Minimal effect	No effect	No data	No effect
Diltiazem	No effect	Use with caution^a^	Use with caution^a^	No data
Carvedilol	No effect	Minimal effect	No data	No data
Felodipine	Minimal effect	Use with caution^a^	No data	No data
Prazosin^d^	Avoid	Avoid	Avoid	Avoid

^a^“Use with caution” indicates that the effect on DOAC exists but does not require dose adjustment unless another interaction is present (concomitant use of other medication with additive interaction or CrCl 30–50 mL/min)

^b^European package insert and European Rhythm Association Practical Guide recommend using 110 mg dose of dabigatran

^c^American package insert does not require dose reduction

^d^Although the prescribing informations recommend that DOACs not to be administered in conjunction with the P-gp inducer like rifampin, studies have not been conducted with other P-gp-inducing medications like prazosin and, therefore, should be avoided, pending the availability of additional data

## Comparative efficacy and safety

In general, the clinical efficacy of DOACs in the four large clinical trials, enrolling over 70 thousand patients, was similar to warfarin [2–7, 29–34]. The “statistical evidence of superiority” of dabigatran over warfarin to prevent ischemic stroke relied on a very tight margin of 0.98 for 95 % confidence interval [0.76 (0.60–0.98), *p* = 0.03]. If one realizes that over 18 thousand patients were necessary to achieve this difference, the practical meaning of this superiority seems to be rather elusive. The real advantage of these new agents is the improved safety margin, particularly evident for apixaban and edoxaban.

### General trial characteristics

To comprehend the combined results of these randomized trials, it is important to review the differences between studies (Table 3). The RE-LY study [2] was an open, blinded endpoint study that compared blinded doses of dabigatran 110 or 150 mg twice daily to open-label dose-adjusted warfarin. Local investigators were not blinded as to which drug was administered. The other three trials were double blinded, double-dummy trials comparing a once daily 20 mg dose of rivaroxaban (ROCKET AF) [3], twice daily, 5 mg dose of apixaban (ARISTOTLE) [4], and two doses (30 and 60 mg) of edoxaban, once daily (ENGAGE AF-TIMI) [5], respectively, to warfarin.

**Table 3:** Characteristics of phase III clinical trials of direct oral anticoagulants

Characteristics	RE-LY (dabigatran)	ROCKET AF (rivaroxaban)	ARISTOTLE (apixaban)	ENGAGE AF (edoxaban)
Design	Randomized, open label^a^	Randomized, DB/DD	Randomized, DB/DD	Randomized, DB/DD
Dosing	150 mg, 110 mg twice daily	20 mg daily	5 mg twice daily	60 mg, 30 mg daily
Dose adjustment/criteria	No	If CrCl 30–49 mL/min then 15 mg	If ≥2 factors: age ≥80 years, body weight <60 kg, creat ≥1.5 mg/dL then 2.5 mg	If CrCl 30–50 mL/min or weight ≤60 kg or potent P-gp inhibitor^b^ then 50 % dose
CrCl exclusion	30 mL/min	30 mL/min	25 mL/min	30 mL/min
CHADS_2_ score inclusion criteria	≥1	≥2	≥1	≥2
Primary efficacy endpoint	Stroke/TIA and SE	Stroke/TIA and SE	Stroke/TIA and SE	Stroke/TIA and SE
Primary safety endpoint	Major bleeding	Major plus CRNM bleeding	Major bleeding	Major bleeding
Trial size	18,113	14,264	18,201	21,105
Age (years), median (IQR)	72 ± 9^c^	73 (65–78)	70 (63–76)	72 (64–78)
CHADS_2_ (mean)	2.1	3.5	2.1	2.8
CHADS_2_ ≥3 (%)	32	87	30	53
Heart failure	32	62	35	57
Stroke/TIA or SE	20^d^	55	19	28
Median follow-up (years)	2.0	1.9	1.8	2.8
Early discontinuation				
DOAC (%)	20.7/21.2	35.4	25.3	33.0/34.3
VKA (%)	16.6	34.6	27.5	34.4

*CRNM* clinically relevant non-major bleeding, *DB/DD* double blind, double dummy, *IQR* interquartile range, *DOAC* direct oral anticoagulant, *SE* systemic embolism, *TIA* transient ischemic attack, *VKA* vitamin K antagonist, *CrCl* creatinine clearance

^a^Patients were unblended with respect to dabigatran or warfarin assignment; however, all investigators, coordinating center members, the steering committee, the event adjudication committee, and the sponsor were blinded during event ascertainment and analyses

^b^Strong P-gp inhibitors such as dronedarone, quinidine, or verapamil

^c^Mean ± SD

^d^No data on SE

While doses of dabigatran were fixed, Xa inhibitor trials incorporated pre-defined criteria for dose reduction at randomization (Table 3). The ENGAGE-AF investigators also employed criteria for post-randomization edoxaban dose modifications. Edoxaban dosing, depending on the treatment arm and clinical characteristics, ranged from 60 to 15 mg daily [2–5].

Patients with severe renal failure were excluded from all of these trials. Each trial had pre-specified CHADS_2_ scores for inclusion. The ROCKET AF and ENGAGE AF trials recruited patients with CHADS_2_ score ≥2. Inclusion criteria for RE-LY and ARISTOTLE included scores ≥1 [2–5].

The primary efficacy endpoint (stroke/TIA and systemic embolism) was identical for all four trials. The principal safety endpoint was major bleeding defined by the International Society for Thrombosis and Haemostasis (ISTH) criteria for all trials. The ROCKET AF trial included a combination of major, plus clinically relevant non-major bleeding [2–5].

Patients were followed for nearly 3 years in the ENGAGE AF trial and for about 2 years in the other three trials.

The median time spent within the therapeutic range for the warfarin arm was the highest in ENGAGE AF and the lowest in the ROCKET AF trial. The impact of warfarin management on the comparative analysis of DOACs efficacy and safety is discussed separately below.

### Trial population characteristics

Differences in NVAF patient inclusion criteria, mainly CHADS_2_ score, resulted in significant differences in clinical characteristics of the recruited populations (Table 3). These differences should be kept in mind when comparing thromboembolic and bleeding rates between studies. The mean CHADS_2_ score was higher in ROCKET AF compared to RE-LY and ARISTOTLE trials. The mean CHADS_2_ score was intermediate in the ENGAGE AF population. Nearly 90 % of participants in the ROCKET AF trial and 53 % of ENGAGE AF participants had a CHADS_2_ score ≥3. In contrast, slightly less than one third of RE-LY and ARISTOTLE trial participants had CHADS_2_ scores of similar severity. ROCKET AF and ENGAGE trials had the highest proportion of patients with CHF (about 60 %) compared to about one third in the other two trials. More than half of ROCKET AF patients had a history of prior stroke. By comparison, prior stroke was present in only 20–30 % of patients in the other three trials [2–5].

There are several practical implications of these differences worth considering. First, these study population differences limit inter-trial outcome comparisons. Neither efficacy nor safety of one agent can be indirectly compared to another. This is particularly true for rivaroxaban and the high CHADS_2_ scores of ROCKET-AF. Second, meta-analyses must take into account differences in patient risk characteristics to be useful for clinical application. Third, in low risk patients (CHADS_2_ ≤2), clinicians can apply the results directly from RE-LY and ARISTOTLE.

Although dose adjustment was allowed at randomization in all three Xa inhibitor trials, practical application of these rules was quite different; only 5 % of ARISTOTLE trial participants had their dose reduced, compared to 21 % of patients in ROCKET AF, and 25 % of patients in the ENGAGE AF study. This indicates that dose adjustment of rivaroxaban and edoxaban was much better explored than apixaban, and this information should be discussed with the patient while deliberating on the choice of a DOAC for someone who would require dose modification. A post hoc analysis of RE-LY data showed that using 110 mg dose of dabigatran for NVAF patients ≥80 years of age or treated with verapamil (dose adjustment consistent with European label) further improved its overall net clinical benefit [35]. While this concept of “tailored dosing” for individual patients is attractive, the lack of direct trial data for dabigatran dose adjustments decreases the validity of this approach. Moreover, available formulations of dabigatran limit the applicability of this concept in the USA.

Co-administration of aspirin was allowed in all four clinical trials. The highest proportion of study participants taking aspirin was in ROCKET AF trial (35 %), followed by ENGAGE AF (29 %), ARISTOTLE (24 %), and RE-LY (21 %) trials. But the latter was the only study that allowed recruitment of patients on clopidogrel (5 % of participants) [2–6, 33]. The proportion of patients taking antiplatelet agents impacts the bleeding rate and needs to be included into any comparative analysis of safety outcomes. Furthermore, this may impact the decision of which anticoagulant to use for patients who require concurrent antiplatelet therapy.

### Individual effectiveness and safety in relation to warfarin

The results of the DOAC trials are generally reported from an intention to treat perspective. From a trialists viewpoint, analyzing and reporting the results from an “intension to treat” perspective is statistically correct, ethically fair, and methodologically pure. Yet, in the end, the on-treatment event rates are what really matters, both to patients and practitioners alike, because it informs what happens when the medication is actually taken. From a patient perspective, an intention to treat analysis reflects information on what would happen if a patient is prescribed a DOAC, regardless of compliance.

The higher treatment discontinuation rates in the ROCKET AF and ENGAGE AF trials (over 30 %), compared to RE-LY and ARISTOTLE trials (over 20 %), analyzed with an intention to treat approach, would negatively impact thromboembolic rates for the former trials. This effect is compounded by the higher stroke risk population in ROCKET AF and ENGAGE AF, as well as the longer follow-up period in ENGAGE AF [2–6]. The effect of a higher, premature discontinuation rate of rivaroxaban and edoxaban on their relative efficacy can be appreciated by an on-treatment analysis. This approach shows superiority over warfarin for both rivaroxaban (HR 0.79, 0.66–0.96; *p* = 0.02) and edoxaban 60 mg (HR 0.79, 0.63–0.99; *p* = 0.002) for prevention of stroke and systemic embolization [7]. By the same reasoning, however, the higher discontinuation rates would favorably impact bleeding rates of rivaroxaban and edoxaban.

In the ENGAGE AF-TIMI 48 study, NVAF patients with creatinine clearance >95 mL/min had an increased rate of ischemic stroke with edoxaban 60 mg once daily compared to patients treated with warfarin. Therefore, the FDA restricted the use of this anticoagulant to those with creatinine clearance lower than 95 mL/min [13]. This restriction seems to have rather limited practical consequences as few NVAF patients have such a high creatinine clearance.

For prevention of systemic embolism only, rivaroxaban was superior to warfarin (in the “as treated” population, see Table 4) and the other DOACs were non-inferior or have no available data (dabigatran). Accordingly, for NVAF patient with the history of systemic embolization, rivaroxaban might be the preferred agent.

**Table 4:** Clinical outcomes of clinical trials with direct oral anticoagulants (DOACs) in relation to warfarin

DOAC vs VKA HR (95 % CI)	RE-LY^a^ (dabigatran) 110 mg	ROCKET AF (rivaroxaban) 20 mg	ARISTOTLE (apixaban) 5 mg	ENGAGE AF-TIMI 48 (edoxaban) 30 mg
	150 mg			60 mg
Ischemic stroke	1.11 (0.89–1.40)^a^	0.94 (0.75–1.17)	0.92 (0.74–1.13)	1.41 (1.19–1.67) p < 0.001
	*0.76 (0.60–0.98)* ^*a*^ *p = 0.03*			1.00 (0.83–1.19)
Systemic embolism	Not reported	*0.23 (0.09–0.61) p = 0.003*	0.87 (0.44–1.75)	1.24 (0.72–2.15)
				0.65 (0.34–1.24)
Hemorrhagic stroke	*0.31 (0.17–0.56) p < .0001*	*0.59 (0.37–0.93 p = 0.024)*	*0.51 (0.35–0.75) p < 0.001*	*0.33 (0.22–0.50) p < 0.001*
	*0.26 (0.14–0.49 p < 0.001*			*0.54 (0.38–0.77) p < 0.001*
Major bleed	*0.80 (0.69–0.93) p = 0.003*	1.04 (0.90–1.20)	*0.69 (0.60–0.80) p < 0.001*	*0.47 (0.41–0.55) p < 0.001*
	0.93 (0.81–1.07) *p* = 0.3			*0.80 (0.71–0.91) p < 0.001*
Intracranial bleed	*0.31 (0.20–0.47) p < 0.001*	*0.67 (0.47–0.93) p = 0.02*	*0.42 (0.30–0.58) p < 0.001*	*0.30 (0.21–0.43) p < 0.001*
	*0.40 (0.27–0.60) p < 0.001*			*0.47 (0.34–0.63) p < 0.001*
Gastrointestinal bleed	1.10 (0.86–1.41)	3.2 vs 2.2^**b**^ p < 0.001	0.89 (0.70–1.15)	*0.67 (0.53–0.83) p < 0.001*
	1.50 (1.19–1.89) p < 0.001			1.23 (1.02–1.50) p = 0.03
All-cause mortality	0.91 (0.80–1.03)	0.85 (0.70–1.02)	*0.89 (0.80–0.98) p = 0.047*	*0.87 (0.79–0.96) p = 0.006*
	*0.88 (0.77–1.00) p = 0.051*			0.92 (0.83–1.01)
Cardiovascular mortality	0.90 (0.77–1.06)^a^	0.89 (0.73–1.10)	0.89 (0.76–1.04)	*0.85 (0.76–0.96) p = 0.008*
	*0.85 (0.72–0.99)* ^*a*^ *p = 0.04*			*0.86 (0.77–0.97) p = 0.013*

Italic font indicates significantly better result of DOAC in relation to warfarin. Bold font indicates significantly worse result of DOAC compared to warfarin

^a^RE-LY reported relative risk instead of hazard ratio (HR); ischemic or uncertain stroke instead ischemic stroke, and vascular mortality instead cardiovascular mortality

^b^Incidence/year (%), HR not reported

The principal efficacy outcome, used in all four trials, was a combination of stroke and systemic embolization that included the safety element of hemorrhagic stroke. This not only double counted medication effect and overestimated the net benefit of the DOACs but also provided misleading information. Proponents would argue that patients do not distinguish stroke types and might tend to lump hemorrhagic and ischemic strokes together. However, this endpoint may be deceptive when it comes to patient care. For the patient with a very high risk of thromboembolism and very low risk of bleeding, the DOAC with the best ischemic rather than global stroke reduction would be desired. In this case, only dabigatran at the higher dose of 150 mg twice a day was superior to warfarin to prevent ischemic stroke (Table 4). The lower dose of edoxaban, 30 mg, was the only DOAC that was inferior to warfarin. All other DOACs and other doses were non-inferior to warfarin [2–6].

The benefit-to-risk ratio of dabigatran 150 mg vs warfarin was less favorable in NVAF older than 75 years compared to younger individuals [33, 35, 36]. For this reason, dabigatran dose of 110 mg would be more appropriate for older patients. However, this dose of dabigatran is not approved in the USA. For elderly patients, the net benefit likely favors one of the factor Xa inhibitors.

Apixaban and both doses of edoxaban were associated with a significantly lower rate of major bleeding, while all the other DOACs were non-inferior to warfarin. All DOACs, except dabigatran dose of 150 mg, were associated with significantly lower rates of fatal bleeding. Hemorrhagic stroke rate and intracranial bleeding were significantly lower in all DOACs, at all doses, compared to warfarin. Only low dose edoxaban demonstrated a lower rate of gastrointestinal bleeding while dabigatran dosed at 150 mg, rivaroxaban, and edoxaban at 60 mg showed a significantly higher rate of gastrointestinal bleeding compared to the VKA arm; apixaban and dabigatran at 110 mg showed the same bleeding rate as warfarin [2–6].

Patients randomization to either apixaban or edoxaban experienced improved overall survival rates relative to warfarin-treated patients. A trend toward improved survival was noted for dabigatran and rivaroxaban. Cardiovascular death rates were significantly lower for dabigatran and edoxaban-treated patients [2–6].

### Comparison of DOACs amongst themselves

The meta-analyses of DOACs as a whole group showed the following: better protection from stroke and systemic embolism, better safety from intracerebral hemorrhage, all-cause mortality, and vascular mortality compared to warfarin. Major bleeding and gastrointestinal bleeding rates were similar to warfarin [37, 38]. This global comparison to warfarin provides an endorsement for DOACs, but has minimal practical usefulness, as the individual patient will take just one agent of this group.

Several indirect, comparative analyses [31, 39–41] amongst DOACs were performed to identify agents with superior efficacy or safety from among the group. Such comparison, however, is seriously impacted by the differences in trial design, patient characteristics, and methods of outcome measurement [7, 42]. To compensate for these differences between trials, only NVAF patients with CHADS_2_ ≥3 were evaluated in one study [30]. This analysis showed that therapy with both doses of dabigatran, apixaban, and rivaroxaban had similar efficacy, but apixaban therapy was associated with a lower rate of major hemorrhage compared to dabigatran 150 mg and rivaroxaban. Very similar efficacy of dabigatran 150 mg, apixaban, and rivaroxaban were also reported when only NVAF patients with prior stroke (secondary prophylaxis) were analyzed [39].

In summary, indirect comparisons of DOACs, adjusted for patient characteristics, provide some meaningful additional information about these agents in the absence of direct comparisons, which are unlikely to be forthcoming. The reader must cautiously interpret these data given the methodological flaws associated with such comparisons.

## Limitations of trials

Numerous exclusion criteria used in all four clinical trials of DOACs left clinicians with a considerable gap of knowledge about applicability of DOACs in important and commonly seen medical conditions. Patients with disabling stroke within the previous 6 months were excluded from dabigatran and rivaroxaban studies. The trial with apixaban excluded patients who suffered ischemic stroke within the previous 7 days; dabigatran and rivaroxaban within the previous 14 days, and edoxaban within the past 30 days. We have no data for DOACs used in patients with prior intracranial, intraocular, spinal, retroperitoneal or traumatic intra-articular bleeding, and in patients with hemoglobin Hb <10 g/dL or a platelet count <100,000 because they were excluded from all four clinical trials [2–5]. Adequate and well-controlled studies of DOAC use in pregnancy and pediatric populations are not yet available.

All four clinical trials showed lower risk of hemorrhagic stroke and intracranial bleed from DOAC therapy compared to warfarin [2–5, 42]. These results are dependent not only on DOACs “performance” but also warfarin arm safety data. Observational “real life” studies confirmed this favorable comparison [43, 44]. However, the “real life” risk of cerebral hemorrhage associated with warfarin [45], documented in a large Canadian registry, was significantly lower than the rate observed in the VKA arms of the DOAC clinical trials. These results, although obtained in a different cohort and clinical setting, might question the “undisputable superiority” of DOACs over warfarin for the risk of intracranial hemorrhage implied in the phase III clinical trials.

Dabigatran, used both for patients with NVAF and venous thromboembolism [46], was associated with increased risk of myocardial infarction. Additional analysis of the RE-LY study outcomes ordered by FDA [47] revealed 81 additional events in the study population including 4 clinically evident and 28 silent myocardial infractions (new Q waves on EKG). This study confirmed a trend toward an increased incidence of myocardial infarction in patients receiving dabigatran but the difference was no longer statistically significant both for 110 and 150 mg doses. Moreover, post-marketing investigations and clinical registries [43, 44] have not substantiated an increased rate of myocardial infarction in NVAF patients treated with dabigatran. It also needs to be highlighted that treatment with dabigatran resulted in significantly lower rates of cardiovascular death compared to warfarin [2].

Difficulty achieving therapeutic anticoagulation, dietary modifications, the necessity of blood collection, visits for INR assessment, and treatment counselling were among important factors responsible for suboptimal use of warfarin in patients with atrial fibrillation. Free of all these inconveniences, DOACs give hope for better compliance with oral chronic anticoagulation in patients with NVAF. However, the rate of discontinuation in all phase III trials was roughly the same for warfarin and DOACs. This suggests that adherence to DOACs is disheartening and might not be as good as expected. However, except for RE-LY, all other studies were double-dummy, so all patients were equally inconvenienced by blood testing and counselling. A “real life” assessment of adherence to DOAC therapy is needed to verify this expectation.

## Controversies related to DOACs

### DOACs reversal

For patients taking warfarin who are found to have prothrombin time-INR prolongation, reversal can be accomplished with fresh frozen plasma, prothrombin complex concentrate (PCC), and vitamin K. Although this approach has a long track record with general endorsement by medical professionals, there is little data showing clinical outcomes of “reversed” to “non-reversed” bleeders. Recently, reported lack of clinical benefit in warfarin-associated intracranial hemorrhage after anticoagulation reversal with PCC calls into question the clinical significance of this therapy [48]. Also, a study comparing patients with intracranial bleeding who were treated with dabigatran, and therefore not “reversed,” compared to those with warfarin, for whom reversal of anticoagulant therapy was possible, did not show improved clinical outcomes with reversal [49]. Moreover, clinical outcome data of bleeding patients on apixaban, and those on warfarin, suggests indirectly that warfarin reversal may not be clinically beneficial [50].

PCC, highly purified concentrates of clotting factors, have been touted as potential reversal agents for patients taking oral direct factor Xa inhibitors. These concentrates are available as either three-factor or four-factor formulations. The four-factor formulation contains factors II, VII, IX, and X in addition to protein C and S. By comparison, the three-factor formulation does not contain factor VII, protein C, or protein S. In one study, a four-factor PCC was assessed in 12 healthy individuals given rivaroxaban 20 mg twice daily or dabigatran 150 mg twice daily. In this study, PCC was shown to normalize prolonged prothrombin times in those patients taking rivaroxaban, but not dabigatran [51]. In another double-blind, randomized, placebo-controlled, two-way crossover study, normal volunteers were given edoxaban 60 mg followed by four-factor PCC (10, 25, or 50 IU/kg) to determine impact on bleeding duration following skin punch biopsy [52]. In this study, a dose-dependent reversal of edoxaban’s effects on bleeding duration, endogenous thrombin potential, and prothrombin time reversal were observed with complete reversal noted at the highest PCC dose. In a separate healthy volunteer study, the three-factor PCC was compared to the four-factor PCC for reversal of rivaroxaban 20 mg twice daily [53]. In this study, only minimal normalization of the prothrombin time was achieved with either PCC; however, the three-factor PCC provided greater changes in thrombin generation. Recently, three-factor PCC was also evaluated for the ability to reverse the anticoagulation effects of edoxaban [54]. This study showed that although there was no apparent reversal of prothrombin time prolongation with three-factor PCC, endogenous thrombin potential was completely reversed. In summary, both the three-factor and four-factor PCCs likely work to some degree for reversal of the direct factor Xa inhibitors. Neither agent is likely to be effective for reversal of dabigatran. Based on our experience, we suggest judicious use of these agents given the propensity for thrombus induction.

Implementation of an antidote, defined as a substance that “neutralizes” or blocks the anticoagulant agent without changing any other components of the coagulation system, should theoretically limit the prothrombotic effect of its use. Several antidotes for DOACs such as idarucizumab, andexanet alfa, and aripazine have shown instantaneous or rapid normalization of coagulation measures in healthy volunteers and are currently evaluated in phase III clinical trials [55]. Recently, the safety of 5 g of intravenous idarucizumab was assessed in patients suffering a serious bleed or requiring an urgent procedure. This study showed that normalization of prolonged clotting tests could be accomplished in 88 to 98 % of the patients within minutes of administration [56]. Based on these data, idarucizumab has now been FDA approved for this indication.

Under circumstances of active bleeding or urgent/emergent surgery, management of these agents would ideally be guided by knowledge of circulating drug levels. Circulating drug levels can be measured directly or indirectly by assessing their impact on clot-based assays. Direct measurement of circulating drug levels is currently limited to academic medical centers. The dilute thrombin time assay supplemented by a specific, validated calibrator allows an indirect measurement of dabigatran plasma levels. Available data [57, 58] indicate that there might be increased bleeding risk if drug concentration at trough is >200 ng/mL. It was also reported [57] that prolongation of aPTT at trough exceeding two times the upper limit of normal range might be associated with excess bleeding risk. On the other hand, a normal aPTT in patients treated with dabigatran was used in a case of an urgent surgery to exclude any relevant residual anticoagulation effect of this DOAC [57]. Similarly, direct Xa inhibitors plasma concentration can also be measure by anti-FXa chromogenic assays using validated calibrators, but no data on threshold values for bleeding or thrombosis yet exist to apply this information into decision making [20]. Wide dissemination of these assays to general practice settings is therefore of principal importance in the effort to gain experience and achieve clinical applicability.

### Labile INR and the relative effectiveness of DOACs

Relevant quality outcomes for anticoagulated patients include the frequency of hard events such as major bleeding and thromboembolism. For warfarin-managed patients, time spent within the therapeutic range is a well-established surrogate outcome which directly correlates with the stroke risk. The correlation with bleeding is variable [59]. The DOAC trials, like all previous multi-center, multinational trials of anticoagulation, showed wide variations in INR control between countries and sites. This has led to questions regarding the relevance of the overall findings for individual patients, or countries, with more refined anticoagulation management systems.

There is an assumption that poor management of warfarin therapy would favor both the efficacy and safety of DOACs and, conversely, more time spent within the therapeutic range would be associated with the loss of benefit of new anticoagulants. The interpretation of the relationships between time in therapeutic range (TTR) and treatment effect is complex and requires digestion before drawing firm conclusions. INR control during warfarin treatment is influenced by multiple patient-related factors such as age, sex, body weight, smoking, diabetes mellitus, liver failure, congestive heart failure, lung disease, prior experience with anticoagulation, and concomitant use of other medications, particularly amiodarone or dronedarone [59]. Other individual-related features, like cultural factors and education, significantly impact anticoagulation management. Furthermore, differences in socioeconomic status, healthcare systems, and quality of medical service have profound impact on the efficacy of anticoagulation management. All these factors have an impact not only on warfarin management, and therefore TTR, but also on efficacy and safety of DOACs. Blinded randomization should account for all of these variables, which are anticipated to be equally prevalent in both treatment arms. Redistributing study results based on individual TTR in the warfarin arm leads to redistribution of younger, better educated patients, with less comorbidities and greater access to sophisticated healthcare systems, into the cluster with better TTR values. Accordingly, the lower risk of stroke and bleeding becomes associated with better warfarin management. However, this “correction” does not happen in the DOAC arm. Thus, using individual TTR to adjust the effect of treatment violates principles of clinical and statistical analysis and annuls the advantages of randomization.

Analyses of the impact of TTR on comparative efficacy and safety of dabigatran [60], rivaroxaban [61], and apixaban [62] were performed using center-specific TTR, which better conserves a fair distribution of factors influencing treatment performance in both arms of the trial. This approach showed that the primary endpoints of efficacy and safety, in relation to warfarin, were consistent across a wide range of center specific TTRs for dabigatran, rivaroxaban, and apixaban [60–62]. Apixaban showed similar efficacy and safety compared to warfarin across both center average TTR and individual TTR quartiles [62].

Taken together, these findings provide no clear evidence for an augmented net clinical benefit of DOACs among patients and populations with poor INR control during warfarin therapy. Conversely, it shows no clear evidence for the loss of benefit (and even potential harm) if replacing good INR management with DOACs. At our institution, we have been fortunate to consistently deliver high TTR for our warfarin-managed patients, and yet, the use of DOAC continues to rapidly expand.

## Choosing a specific antithrombotic agent

### To anticoagulate or not anticoagulate

The first step in this decision making process is to determine whether the patient requires an anticoagulant. The landscape of antithrombotic decision making for NVAF is rapidly evolving due, in part, to the introduction of CHA_2_DS_2_-VASc. This scoring tool qualifies more patients for anticoagulant therapy who were previously deemed low risk by the CHADS_2_ scoring tool [63, 64]. In the past, anticoagulant therapy would have been recommended for 66 % of patients with NVAF [64]. Using this new scoring system, between 90 and 95 % of patients will qualify for anticoagulant therapy [65, 66]. The second major advance altering this landscape is the low bleeding rates associated with DOAC therapy. In the current climate, the vast majority of NVAF patients should be offered anticoagulant therapy. The question is not who should receive anticoagulant therapy, but rather who should not. Identifying the rare patient who is best served without antithrombotic therapy includes those with active bleeding, recurrent anticoagulant related bleeding, and those with a CHA_2_DS_2_-VASc score of 0. If the only CHA_2_DS_2_-VASc variable is female gender, then these patients also do not require anticoagulant therapy. If a NVAF patient had major bleeding on warfarin, anticoagulation often should not be completely eliminated as the therapeutic option until apixaban, or lower dose of dabigatran, or edoxaban is tried.

The traditional warfarin alternative for NVAF patients who are not suitable, not willing, or not requiring anticoagulation, has been aspirin. Compared to aspirin in the AVERROES trial, apixaban showed a similar major bleeding (1.4 vs 1.2 %, *p* = 0.57) and intracranial bleeding rates (11 cases with apixaban vs 13 with aspirin), but significantly better protection from stroke (1.6 vs 3.7 %, *p* < 0.001) [6]. These data suggest that antiplatelet therapy should not be offered for NVAF unless the patient refuses an anticoagulant or cannot afford apixaban [16, 20].

### Tailoring anticoagulant choice

The next step in the decision making is to identify patient-specific factors, which would help tailor the anticoagulant choice (Table 5). For patients at increased risk of thromboembolism with acceptable bleeding risk, we prefer dabigatran 150 mg twice daily. This is the only antithrombotic agent shown to have superior efficacy in the reduction of ischemic stroke. The superiority rating noted for apixaban included a reduction in hemorrhagic stroke in the composite outcome. Therefore, for relatively young patients with good kidney function and no history of bleeding, but with the presence of left atrial appendage thrombus, dabigatran appears to be the best option. Apixaban does not offer a benefit over warfarin for this patient profile, as the risk of bleeding, including intracranial bleeding, is relatively low. In this case, edoxaban 30 mg will be the least attractive option as it is the only DOAC with higher ischemic stroke rates compared to warfarin.

**Table 5:** Clinical situation related preferences for the use of direct oral anticoagulants

Clinical situation	First choice	Second choice	Avoid
High thromboembolic and low bleeding risk	Dabigatran 150 mg	Apixaban, edoxaban 60 mg, rivaroxaban, dabigatran 110 mg	Edoxaban 30 mg
Low thromboembolic and high bleeding risk	Edoxaban 30 mg Apixaban	Edoxaban 60 mg Dabigatran 110 mg	Dabigatran 150 mg Rivaroxaban
Moderate thromboembolic and bleeding risk	Apixaban Edoxaban 60 mg Dabigatran 110 mg	Rivaroxaban Dabigatran 150 mg	Edoxaban 30 mg
High thromboembolic and bleeding risk	Apixaban	Rivaroxaban Edoxaban 60 mg Dabigatran 150 mg	Edoxaban 30 mg
Compliance concerns	Edoxaban 60 mg Rivaroxaban^a^	Edoxaban 30 mg	Dabigatran or apixaban
Moderate renal dysfunction^b^	Apixaban	Rivaroxaban Dabigatran 110 mg Edoxaban 60 or 30 mg	Dabigatran 150 mg

^a^Although dosing instruction recommends taking rivaroxaban with evening meal, in reality it means that it needs to be taken with food either in the morning or in the evening

^b^Creatinine clearance 30–44 mL/min (chronic kidney disease stage 3B). We remain hesitant to recommend any of these agents for CKD stages 4 or 5 until published safety data are available

For patients at increased risk of bleeding, we prefer apixaban whereby this agent provided a consistent reduction in bleeding outcomes regardless of the antithrombotic indication [4, 67]. Edoxaban would be a reasonable alternative choice.

Patients with NVAF and recurrent gastrointestinal bleeding pose a particular challenge. Edoxaban at the reduced dose of 30 mg could be considered in this clinical situation whereas this is the only preparation associated with a lower rate of gastrointestinal bleeding compared to warfarin [5]. Although edoxaban 60 mg is recommended for thromboembolism prevention in NVAF patients, current guidelines encourage a dose reduction in patients with high bleeding risk [17–20]. Dabigatran 150 mg, rivaroxaban, and edoxaban 60 mg should be avoided in these patients because they experienced a higher gastrointestinal bleeding rate compared to warfarin. Dabigatran 110 mg and apixaban were associated with gastrointestinal bleed rates similar to warfarin. Interestingly, dabigatran showed a similar proportion of upper and lower gastrointestinal bleeding, whereas rivaroxaban and apixaban use was associated with upper gastrointestinal bleeding in two thirds of cases [2–4]. It is speculated that high concentrations of active DOACs in feces explains the relatively high gastrointestinal bleeding rate of these medications. It is further postulated that the high concentration of the pro-drug dabigatran etexilate in the colon becomes activated to dabigatran by mucosal esterases, and consequently results in bleeding from this site [7]. For these combined reasons, we favor Xa inhibitors over dabigatran in patients with the prior episodes of lower gastrointestinal bleeding. This is particularly relevant for those patients who have undergone recent polypectomy. For these patients, it would be reasonable to consider an alternate anticoagulant for the several week period of polypectomy site healing.

In the setting of chronic kidney disease, particularly those patients requiring dialysis, warfarin remains the first choice. Apixaban is FDA approved for patients with chronic kidney disease without a recommended dose adjustment regarding of CKD stage. Until further clinical experience is reported, we remain cautious regarding use of this drug for patients with end-stage renal disease.

Once daily dosing has been shown to improve compliance and adherence over medications requiring multiple daily dosing [68]. To promote adherence, we, therefore, would consider rivaroxaban or edoxaban.

For patients with significant dyspepsia, peptic ulcer disease, after vagotomy, gastric drainage procedure, antrectomy, subtotal or total gastrectomy, and after bariatric procedure, we suggest avoiding dabigatran which may increase symptoms of peptic ulcer and/or interfere with medication absorption. Because of limited gastrointestinal absorption of dabigatran (6–8 %), even minor fluctuations may have a profound impact on plasma levels.

Patients who had peripheral embolism as a thrombotic complication of NVAF might be treated preferentially with rivaroxaban as it is the only DOACs with improved efficacy for this type of event.

For patients older than 75 years, we prefer direct Xa inhibitors over dabigatran dose of 150 mg because of unfavorable benefit-risk balance for the latter in elderly.

When treating patients with significant coronary disease, we suggest avoiding dabigatran until more published experience is available.

For those patients who are already well-established on a stable warfarin regimen, there is no need to contemplate a change. An attractive option for streamlining this regimen is the addition of home INR monitoring which has been shown to improve safety, time in the therapeutic range, and patient satisfaction through increased flexibility and more frequent INR assessment [69].

## Conclusion

Atrial fibrillation is commonly encountered in clinical practice, and some form of oral anticoagulation is indicated in almost all patients. Relative to warfarin, the clinical experience with DOACs has been limited and many questions remain. Providers must be familiar with the characteristics of these agents and the trials on which their use was established in order to counsel and care for the growing number of patients taking them. In general, DOACs have shown similar efficacy, with better safety, compared to warfarin for NVAF. This improved safety might further expand the proportion of NVAF patients who would benefit from anticoagulation therapy. Ultimately, patient-specific factors and shared decision making should guide anticoagulant selection.
